# Tropical modulation of East Asia air pollution

**DOI:** 10.1038/s41467-022-33281-1

**Published:** 2022-09-23

**Authors:** Myung-Il Jung, Seok-Woo Son, Hyemi Kim, Deliang Chen

**Affiliations:** 1 School of Earth and Environmental Sciences, Seoul National University, Seoul, South Korea; 2 School of Marine and Atmospheric Sciences, Stony Brook University, Stony Brook, New York, NY USA; 3 Department of Earth Sciences, University of Gothenburg, Gothenburg, Sweden

**Keywords:** Atmospheric dynamics, Environmental impact

## Abstract

Understanding air pollution in East Asia is of great importance given its high population density and serious air pollution problems during winter. Here, we show that the day-to-day variability of East Asia air pollution, during the recent 21-year winters, is remotely influenced by the Madden–Julian Oscillation (MJO), a dominant mode of subseasonal variability in the tropics. In particular, the concentration of particulate matter with aerodynamic diameter less than 10 micron (PM_10_) becomes significantly high when the tropical convections are suppressed over the Indian Ocean (MJO phase 5–6), and becomes significantly low when those convections are enhanced (MJO phase 1–2). The station-averaged PM_10_ difference between these two MJO phases reaches up to 15% of daily PM_10_ variability, indicating that MJO is partly responsible for wintertime PM_10_ variability in East Asia. This finding helps to better understanding the wintertime PM_10_ variability in East Asia and monitoring high PM_10_ days.

## Abstract (web summary)

In this study, it is suggested that the daily PM_10_ level in East Asia is remotely controlled by the convection over the equatorial Indian Ocean and western Pacific. This tropical modulation explains up to 15% of daily PM_10_ variability in the region.

## Introduction

Severe air pollution events, with high PM_10_ concentration, have frequently occurred in East Asia over the past decades, despite the government’s air quality regulation^1–3^. Such events, which are most frequent in cold season^4^, have often been explained by local emissions and meteorological conditions that control aerosol deposition, accumulation, and transport^5,6^. Although minor, they are also influenced by climate variability on a wide range of time scales. On the interannual time scale, for instance, wintertime PM_10_ concentration in East Asia is weakly sensitive to the El Niño–Southern Oscillation (ENSO)^7^ and Arctic sea ice variability^8,9^. The relationship between haze days and Pacific Decadal Oscillation (PDO)^10,11^ implies that East Asia PM_10_ concentration may be affected by climate oscillation on the decadal time scale. East Asia PM_10_ concentration also substantially varies on the daily time scale. However, its linkage to subseasonal climate variability has received little attention.

The Madden–Julian Oscillation (MJO)^12,13^ is a planetary-scale convection–circulation coupled system in the tropics that typically moves eastward from the tropical Indian Ocean to the western Pacific with a period of 30 to 60 days. As the MJO develops and propagates, it influences the midlatitude circulation and weather events by exciting the Rossby wave train^14,15^. It is reported that the MJO-induced circulation changes can also modulate local air pollutants, such as aerosol and ozone^16–22^. For example, in Santiago, Chile, the dispersion of air pollutants is influenced by the MJO-related atmospheric circulation^20^. Another example is air pollution in South and Southeast Asia. In these regions, air quality is directly modulated by the MJO^16,21,22^. For instance, the variability of PM_10_, CO, and ozone in Malaysia is significantly affected by the MJO^22^.

However, the remote linkage between MJO and PM_10_ concentration in East Asia has not been addressed yet. Since East Asia is on the main pathway of the Rossby wave train, precipitation, temperature, and atmospheric circulations are significantly modulated by the MJO^23–26^. Therefore, we hypothesize that MJO affects local air pollution over East Asia by changing meteorological conditions via tropics–extratropics teleconnections. In this study, we show that daily variability of East Asia PM_10_ concentration is significantly modulated by subseasonal climate variability in the tropics. Specifically, we show that wintertime PM_10_ variability in East Asia is to a large extent determined by the MJO.

## Results

### MJO-related PM_10_ change in East Asia

**Fig. 1:**
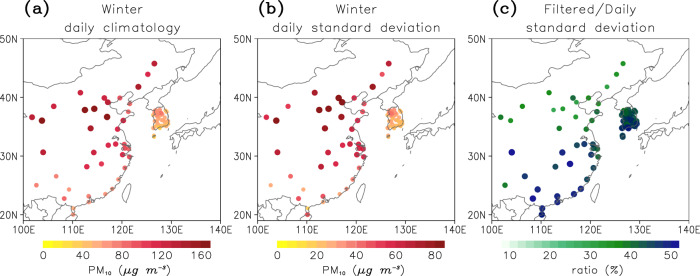
Winter PM_10_ concentration in East Asia. **a** Daily climatology, (**b**) daily standard deviation, and (**c**) the ratio of the 20–96 days filtered subseasonal standard deviation to a daily standard deviation during the boreal winter from 2001 to 2021.

Figure 1a presents PM_10_ climatology in China and Korea during the boreal winter (December-February) from 2001 to 2021. The overall PM_10_ level, ranging from 30 μg m^−3^ to 200 μg m^−3^, is relatively high compared to that in Europe and North America^27^. Urban areas in northern China (including Beijing), where PM_10_ concentration is typically determined by local emissions of primary pollutants and their regional transports, show higher values than rural areas in southern China where PM_10_ concentration is largely attributable to the dusts from agricultural sources and transportations^28^. Most monitoring stations in Korea exhibit relatively low PM_10_ concentration than those in China, but megacities such as Seoul still show a high PM_10_ concentration.

The daily PM_10_ variability is quantified in Fig. 1b by computing daily standard deviation. The daily standard deviation of station-averaged PM_10_ concentration is 52 μg m^−3^, which accounts for 54% of the daily climatology (~96 μg m^−3^), indicating a significant day-to-day variability of East Asia PM_10_ concentration, especially at the stations with high PM_10_ climatology in northern China. Approximately 30% to 50% of this daily variability is due to subseasonal variability which is quantified by a 20–96 days band-pass-filtered PM_10_ anomaly (Fig. 1c).

**Fig. 2:**
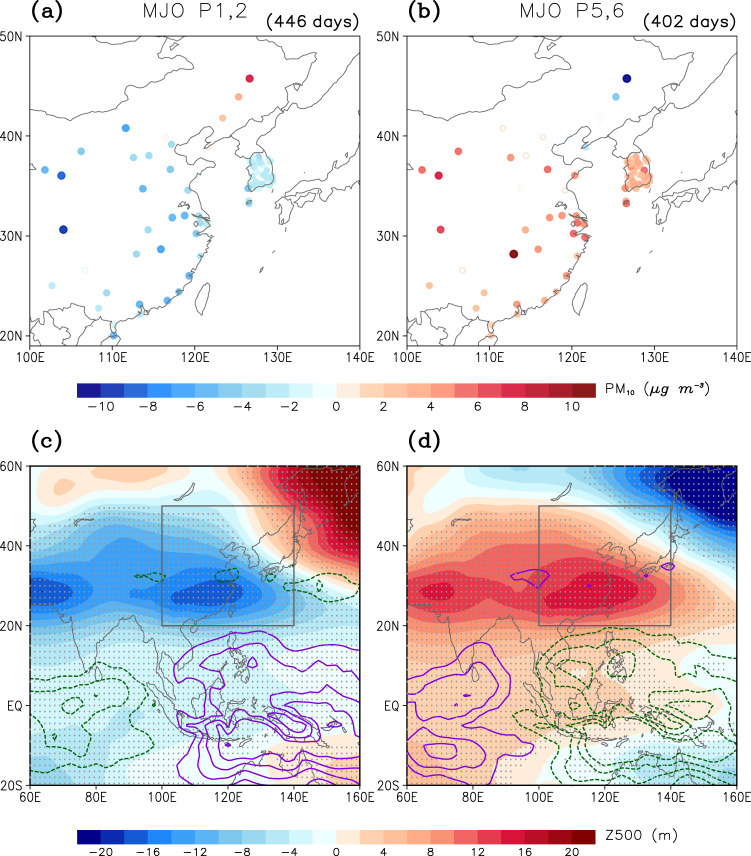
Madden–Julian Oscillation (MJO)-related PM_10_, 500-hPa geopotential height (Z500), and outgoing longwave radiation (OLR) anomalies. Composite PM_10_ anomalies averaged over lag 6–10 days during Madden–Julian Oscillation (MJO) phases (**a**) 1–2 and (**b**) 5–6. Multiple counts of the same day are not allowed. Sample size is denoted at the top-right corner, and values that are statistically significant at the 95% confidence level are denoted with filled circles. **c**, **d** Same as (**a**, **b**) but for anomalies of 500-hPa geopotential height (Z500) (shading) averaged over lag 6–10 days and tropical outgoing longwave radiation (OLR) (purple/green contours represent positive/negative values with 5 W m^−2^ interval) at lag 0 day from 20°S to 60°N. Values that are statistically significant at the 95% confidence level are dotted. Gray boxes are the analysis domains shown as (**a**, **b**).

The PM_10_ concentration in East Asia can be regulated not only by local emissions but also by atmospheric conditions^5–7,29^. On the subseasonal time scale, midlatitude circulations are substantially influenced by remote processes. One of the most important remote processes that affect the atmospheric circulations in East Asia is the tropical–extratropical teleconnections driven by the MJO^14^. By exciting the Rossby gyres in the subtropics and the Rossby waves that propagate into the midlatitudes^14,15^, MJO effectively modulates the precipitation and atmospheric circulations in East Asia^23–26^. When MJO convection is enhanced over the tropical Indian Ocean and suppressed over the western Pacific (MJO phase 1–2; Fig. 2c), a robust anomalous cyclonic circulation, corresponding to a negative anomaly of 500-hPa geopotential height (Z500), develops over East Asia, especially in southern China, Korea, and western Japan. The opposite is true for the mirror phase of MJO, in which convection is suppressed over the tropical Indian Ocean and enhanced over the western Pacific (MJO phase 5–6; Fig. 2d).

These MJO-related atmospheric circulation anomalies significantly modulate the wintertime PM_10_ concentration in East Asia. Figure 2a, b show PM_10_ anomalies during MJO phases 1–2 and 5–6 at lag 6–10 days (i.e., 6–10 days after the selected MJO phase). This time lag matches with the time scale by which the MJO-induced Rossby waves take to affect in the midlatitudes^30,31^. During MJO phase 1–2, PM_10_ concentration becomes anomalously low almost everywhere except in northeastern China (Fig. 2a). The station-averaged PM_10_ anomaly is approximately −4 μg m^−3^, being statistically significant at the 95% confidence level. In contrast, PM_10_ concentration is anomalously high during MJO phase 5–6 (Fig. 2b) with the station-averaged PM_10_ anomaly of approximately 4 μg m^−3^. Their spatial distribution is almost the mirror image to MJO phase 1–2 (see Supplementary Fig. 1 for the other MJO phases). This result indicates that MJO systematically modulates PM_10_ concentration in East Asia.

Here we emphasize that although the station-averaged PM_10_ anomaly is relatively small compared to the daily standard deviation (i.e., ± 15%), its value at each station can be large. The PM_10_ difference between MJO phases 1–2 and 5–6 and its ratio to the daily standard deviation are evaluated in Supplementary Fig. 2. It turns out that MJO impact is relatively small in the in-land stations where the daily PM_10_ variability is large (e.g., Beijing–Tianjin region). However, its impact is substantially large along the coast. For instance, the MJO-related PM_10_ anomaly in South Korea reaches up to 35% of daily variability. This result suggests that MJO plays a critical role in determining daily PM_10_ concentration in central and southern China as well as in South Korea.

**Fig. 3:**
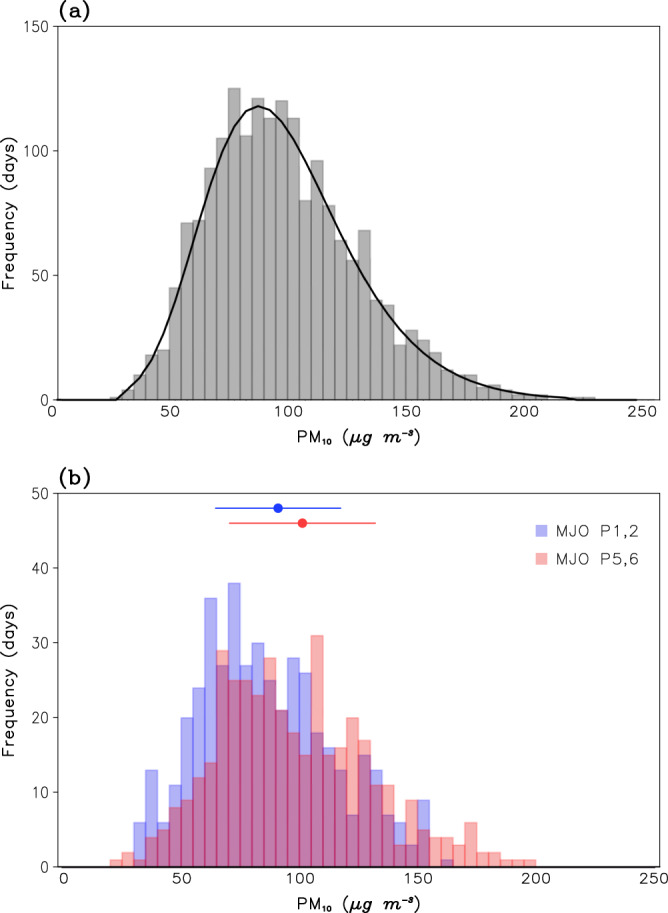
Frequency distribution of station-averaged PM_10_ concentration. **a** Frequency distribution of all PM_10_ observations with a bin size of 5 μg m^−3^. Black line represents the generalized extreme value distribution. Unfiltered data are used. **b** Same as (**a**) but for the PM_10_ from lag 6–10 days during Madden–Julian Oscillation (MJO) phases 1–2 (blue) and 5–6 (red). Mean and one standard deviation are indicated at the top with dot and horizontal error bar, respectively.

The MJO also modulates extreme PM_10_ days. Figure 3 shows the frequency distribution of the station-averaged PM_10_ concentration. Unfiltered data is used here. The frequency distribution exhibits a long tail with increasing PM_10_ concentration (Fig. 3a), indicating that high pollution days occur more frequently than clean air days. A similar frequency distribution, with different mean and extremes, is also found when MJO phases 1–2 and 5–6 are separately considered (Fig. 3b). The daily PM_10_ concentration during MJO phase 1–2 is on average around 89 μg m^−3^, which is 7% smaller than the wintertime average of about 96 μg m^−3^. However, the daily PM_10_ concentration during MJO phase 5–6 is frequently observed at higher values, with its mean value of about 100 μg m^−3^. This is approximately 1.2 times higher than that during MJO phase 1–2. This result is consistent with Fig. 2 which is based on subseasonally-filtered PM_10_ concentration. It is noticeable from Fig. 3b that PM_10_ distribution is skewed toward high pollution days during MJO phase 5–6. High PM_10_ days, defined as days with PM_10_ concentration greater than 140 μg m^−3^ corresponding to the top 10 percentile, are more frequently observed during MJO phase 5–6. This result indicates that MJO teleconnections modulate not only the mean PM_10_ concentration but also the frequency of high PM_10_ days in East Asia.

### How does the MJO modulate East Asia PM_10_?

**Fig. 4:**
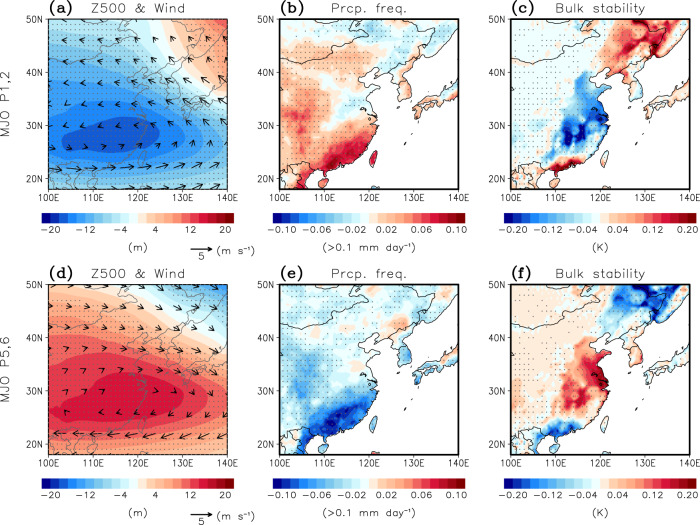
Atmospheric circulation anomalies during Madden–Julian Oscillation (MJO) phases 1–2 and 5–6. Composite anomalies of (**a**) 500-hPa geopotential height (Z500) (shading) and wind (vectors), (**b**) precipitation frequency which is counted as one if the daily precipitation exceeds 0.1 mm day^−1^, and (**c**) bulk stability which is defined as potential temperature difference between 925 hPa and 1000 hPa averaged over lag 6–10 days during Madden–Julian Oscillation (MJO) phase 1–2. **d–f** Same as (**a–c**) but for Madden–Julian Oscillation (MJO) phase 5–6. Dotted areas and black arrows denote statistically significant values at the 95% confidence level.

The MJO-related PM_10_ anomalies likely result from the changes in atmospheric ventilations and precipitation (Fig. 4; see also Supplementary Figs. 3 and 4 for the temporal evolution). The enhanced convection over the tropical Indian Ocean during MJO phase 1–2 yields the subtropical Rossby gyre and excites the Rossby wave train toward midlatitudes^32,33^. They strengthen moisture transport and vertical motion in East Asia, leading to more frequent precipitation events (Fig. 4b). Since precipitation can remove PM_10_ via wet scavenging processes^34^, it could contribute to a decrease in PM_10_ concentration during MJO phase 1–2. As hypothesized, positive precipitation frequency anomalies (Fig. 4b) indeed match well with PM_10_ anomalies, especially in southeastern China (Fig. 2a).

Consistent with previous studies, Z500 shows cyclonic anomaly in the mid-troposphere from eastern China to Korea during MJO phase 1–2 (Fig. 4a). The center of circulation anomaly, which is a part of the Rossby gyre, is associated with updraft that promotes the dispersion of air pollutants^35^. The bulk stability, which is defined by the potential temperature difference between 925 hPa and 1000 hPa, is indeed reduced, especially in eastern China (Fig. 4c). This unstable condition below the planetary boundary layer could reduce PM_10_ concentration through strong vertical ventilation. The combined effect of more frequent precipitation events and enhanced atmospheric ventilations could lead to low PM_10_ concentration in East Asia during MJO phase 1–2.

The anomalous atmospheric condition switches to opposite sign during MJO phase 5–6. Precipitation events become less frequent in East Asia (Fig. 4e), thus contributes to anomalously high PM_10_ concentration (Fig. 2b). However, this may not be sufficient to produce high PM_10_ concentration as a relatively dry condition itself does not guarantee more frequent high PM_10_ days. Other factors such as atmospheric ventilation changes may also play a role^8,36^. The anticyclonic circulation anomaly drives anomalous downward motion that promotes the accumulation of local pollutants^6^. Along with this, the positive anomaly of bulk stability is observed in eastern China (Fig. 4f). The stable boundary layer can lead to reduced vertical ventilation, increasing PM_10_ concentration^8,9^. This together suggests that relatively high PM_10_ concentration in East Asia during MJO phase 5–6 is likely caused by the combined effects of less frequent precipitation events and reduced atmospheric ventilations.

The atmospheric condition changes in response to the MJO are further compared with the daily standard deviation in Supplementary Figs. 5d–f. The MJO-related precipitation change is over 30% of day-to-day precipitation variability in southern China and over 10% in eastern China and South Korea. The bulk stability change also accounts for over 20% of daily variability in eastern China. It is noteworthy that a few stations in northeastern China show the MJO-related PM_10_ anomalies whose sign is opposite to most other stations (Fig. 2). Although the reason is unclear, it is again consistent with the opposite-signed precipitation and near-surface stability responses to the MJO in the region (Fig. 4).

**Fig. 5:**
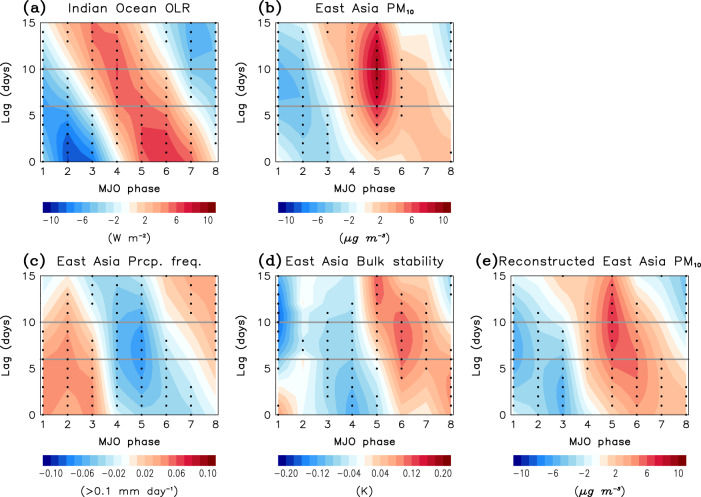
Hovmöller diagram of Madden–Julian Oscillation (MJO) convection and East Asia PM_10_. **a** Outgoing longwave radiation (OLR) anomalies averaged over 60°–80°E and 10°S–10°N and station-averaged anomalies of (**b**) PM_10_ concentration, (**c**) precipitation frequency, (**d**) bulk stability, and (**e**) PM_10_ concentration reconstructed by precipitation frequency and bulk stability with respect to the Madden–Julian Oscillation (MJO) phase and time lag. Values that are statistically significant at the 95% confidence level are indicated with black dots. The gray lines denote the lag 6–10 days which are used in the composite analysis in Figs. 2–4.

The temporal evolution of the MJO convection and its relationship with the station-averaged PM_10_ concentration in East Asia are further demonstrated by considering eight MJO phases in Fig. 5. As shown in Fig. 2, tropical convection is enhanced in the equatorial western Indian Ocean (60–80°E, 10°S–10°N) during MJO phase 1–2 (blue shading at lag 0 day) but suppressed during MJO phase 5–6 (red shading at lag 0 day) (Fig. 5a). These anomalies switch the sign within 15–30 days as the enhanced and suppressed convection couplets move eastward in time across the Maritime Continent. This makes a systematic OLR evolution as shown in Fig. 5a.

Figure 5b shows the temporal evolution of PM_10_ anomaly in East Asia. As anticipated from Fig. 2, it well follows the evolution of the MJO convection (Fig. 5a). Most importantly, the maximum PM_10_ anomaly appears 8–10 days after the maximum OLR anomaly. A large negative PM_10_ anomaly at lag 8 day from the MJO phase 1 is connected to the enhanced convection over the western Indian Ocean. Likewise, a large positive PM_10_ anomaly at lag 10 day from the MJO phase 5 is linked to the suppressed convection over the western Indian Ocean. Since the time interval from one MJO phase to another is about 5–8 days, these PM_10_ anomalies partly include the responses to MJO phases 2 and 6 at a shorter time lag.

The time-lagged remote connections can be again explained by the precipitation and bulk stability changes induced by MJO teleconnections (Fig. 5c, d). Here the grid point closest to each station is used to compute the station-averaged value. Multiple counts are not allowed. The atmospheric circulation anomalies resemble the PM_10_ anomalies. The reduced PM_10_ concentration, followed by MJO phase 1–2, is closely related with an enhanced precipitation frequency and a reduced bulk stability. In contrast, the increased PM_10_ concentration after MJO phase 5–6 is related with a reduced precipitation frequency and an increased bulk stability. The combined effect of precipitation frequency and bulk stability changes in response to the MJO well captures the temporal evolution of East Asia PM_10_ anomalies (Fig. 5e).

## Discussion

This study reveals that the wintertime PM_10_ concentration in East Asia is significantly modulated by the MJO. Specifically, MJO phase 1–2 tends to lead to reduced PM_10_ concentration, presumably due to more frequent precipitation events and strong atmospheric ventilation in East Asia. In contrast, during MJO phase 5–6, PM_10_ concentration becomes anomalously high possibly due to less frequent precipitation events and weak atmospheric ventilation. This finding is all based on the time average from lag 6–10 days (Figs. 2–4), the former lagging the latter. It is anticipated that when MJO phase 5–6 is detected in the tropics, the PM_10_ in East Asia increases with a high chance of heavy pollution days one to two weeks later. Although such a simple relationship does not guarantee the quantitative prediction, it is helpful to better understand the daily variability of East Asia PM_10_ concentration and monitor high PM_10_ days.

Given the high population density and serious air pollution problems in East Asia, any added competency in understanding PM_10_ characteristics is of great significance. Nevertheless, the results presented here need to be interpreted with caution because of several limitations. First, PM_10_ data in China are partly derived from the air pollution index^37^. The data conversion may have introduced small but non-negligible biases. Second, PM_10_ data are available only for 21 years from 2001 to 2021. This is a rather short-term record, compared to long-term meteorological observations commonly used in the MJO teleconnection studies. To better quantify the MJO–PM_10_ relationship, longer data records are required. Third, the effects of local emissions and regional transports of PM_10_ are not fully eliminated in this study. To isolate the MJO–teleconnection impacts, it may be useful to employ numerical modeling in which the local processes can be controlled.

## Methods

### Observations and Reanalysis Data

Daily PM_10_ concentrations are obtained from air quality monitoring stations in Korea and China for 21 years (2001-2021). The Korean PM_10_ data are directly obtained from the Ministry of Environment, Republic of Korea (https://www.airkorea.or.kr), while the long-term record of daily PM_10_ concentration in China is obtained from the two different sources. Until December 2012, PM_10_ concentration is estimated by converting the air pollution index (API)^37^. The unitless API is determined by the maximum pollutant concentration among the three pollutants, i.e., PM_10_, SO_2_, and NO_2_. When the API is 50 or higher, only the PM_10_-based API is used. This accounts for more than 85% of the total analysis period. However, the API smaller than 50 does not report the principal pollutant. In such a case, the API is simply assumed to be derived from PM_10_. This introduces the uncertainty in low PM_10_ concentrations. Despite this uncertainty, the PM_10_ concentration derived from the API is closely related with the observed PM_10_ concentration with a correlation coefficient of up to 0.9^37,38^.

In March 2012, Ministry of Environmental Protection in China announced an official revision from the API to the air quality index (AQI). This index is based on the six pollutants, i.e., PM_10_, PM_2.5_, SO_2_, NO_2_, O_3_, and CO^39–41^. The Air Quality Inspection Platform of China provides the concentration of these six pollutants and the resultant AQI since December 2013 (http://www.aqistudy.cn/historydata/). Therefore, PM_10_ concentration after December 2013 are collected from this website. It may be noted that no data are available in China for 11 months from January to November 2013.

The transition from the API to the AQI in 2013 may introduce the uncertainty in PM_10_ concentration. To test the sensitivity of our result to data sources, all analyses are repeated for the two different periods, i.e., the early period from 2001/2002 winter to 2012 December using the API-derived PM_10_ data, and the later period from 2013/2014 winter to 2020/2021 winter using the AQI-collected PM_10_ data. The MJO-dependent PM_10_ anomalies in these two periods are quantitatively similar (Supplementary Fig. 6), indicating that the MJO-PM_10_ relationship in China is not sensitive to the choice of data sources.

For both the Korean and Chinese stations, only those that exceed 75% of the temporal coverage are considered. This allows inclusion of a total of 100 stations, with 58 in Korea and 42 in China. For the station-averaged PM_10_ concentration, only the stations located below 40°N, which show a consistent response according to the MJO phase, were used (Fig. 1). Because Korean stations are densely distributed, only six stations (i.e., Busan, Daejeon, Gangneung, Gwangju, Jeju, and Seoul) are subsampled in this calculation. The overall results do not change much when different stations are selected.

The daily meteorological variables, including geopotential height, wind, and temperature, are obtained from European Centre for Medium Range Weather Forecasts Reanalysis 5 (ERA5)^42^. The gauge-based precipitation data from the Climate Prediction Center (CPC) (https://ftp.cpc.ncep.noaa.gov/precip/CPC_UNI_PRCP/) and the outgoing longwave radiation (OLR) data from the National Oceanic and Atmospheric Administration (NOAA) (https://psl.noaa.gov/data/gridded/data.olrcdr.interp.html) are also used. All variables are linearly interpolated into a horizontal resolution of 0.5° longitude × 0.5° latitude.

The daily climatology of each variable is derived from a 31-day moving average of the calendar day mean over 21 years. The anomaly is then calculated by subtracting the daily climatology from the raw data. To focus on the subseasonal variability, the low-frequency anomaly is further isolated by applying a 20–96 day Lanczos band-pass-filter to the daily anomaly. The cut-off periods of 20 and 96 days, the frequency associated with the MJO, are determined by the definition of the OLR-based MJO index (OMI)^43^ used in this study. The resultant anomaly, which is referred to as the subseasonal anomaly, is used in the composite analysis. The statistical significance of the composite value is tested using Student’s *t*-test. When calculating the degree of freedom, a continuous timeseries of one MJO phase (e.g., phase 1), separated by at least 7 days from the date of another MJO phase 1, is considered to be one degree of freedom^44^. The null hypothesis is that the population mean is zero.

Only the boreal winter (December to February) is considered in this study. When considering the extended winter, the overall results do not change much although some results become slightly weak. There is also no noticeable difference when detrended data are used.

### MJO index and precipitation frequency index

The OMI^43^ is utilized in this study. This index is directly obtained from NOAA website (https://www.psl.noaa.gov/mjo/mjoindex/). The MJO phase is defined with the two leading principal components (PC) timeseries, which are calculated by projecting 20–96 days filtered OLR anomalies over 20°S–20°N onto the two leading empirical orthogonal functions. Following previous studies, the MJO phase is divided into eight phases, depending on the location of OLR anomalies. The MJO amplitude is defined as below.

1 $$\sqrt{{{{{{{\rm{PC}}}}}}1}^{2}+{{{{{{\rm{PC}}}}}}2}^{2}}$$

Only well-organized MJO days, with the MJO amplitude greater than one, are considered in the composite analysis. Approximately 64% of days in the analysis period are classified as MJO days.

To relate PM_10_ concentration to precipitation events, the precipitation frequency index is defined. It is counted as one if the daily precipitation exceeds 0.1 mm day^−1^, otherwise, it is set to zero. Although not shown, the key results are not sensitive to the choice of precipitation threshold varying from 0.1 to 5 mm day^−1^. It is considered that more frequent precipitation (or a higher precipitation index) is associated with a lower PM_10_ concentration.

### Reconstruction of East Asia PM_10_

East Asia PM_10_ anomaly in each MJO phase (Fig. 5e) is reconstructed by conducting multiple linear regression analysis:

2 $${{{{{{\rm{PM}}}}}}}_{10}^{{\prime} }={{{{{\rm{a}}}}}}\times {{{{{\rm{PF}}}}}}+{{{{{\rm{b}}}}}}\times {{{{{\rm{BS}}}}}}+{{{{{\rm{c}}}}}}$$

where ${{{{{{\rm{PM}}}}}}}_{10}^{{\prime} }$, PF, and BS are PM_10_ anomaly, precipitation frequency, and bulk static stability averaged over East Asia domain, respectively. When values shown in Fig. 5c, d are used, *a* = −81.9 μg m^−3^ and *b* = 21.7 μg m^−3^ K^−1^ with a small residual of *c* = 0.3 μg m^−3^. The variance inflation factor is 1.0.

## Supplementary information

Supplementary Information

## Data Availability

Daily PM_10_ data of Korea used in this study are obtained from the Ministry of Environment, Republic of Korea (https://www.airkorea.or.kr/web/last_amb_hour_data?pMENU_NO=123). Chinese PM_10_ data are collected from the air quality index website (https://aqicn.org/city/) and the Air Quality Inspection Platform of China (https://www.aqistudy.cn/historydata/). The meteorological variables are downloaded from the ERA5 website (https://cds.climate.copernicus.eu/cdsapp#!/dataset/reanalysis-era5-pressure-levels?tab=form). Gauge-based precipitation data from CPC (https://ftp.cpc.ncep.noaa.gov/precip/CPC_UNI_PRCP/) and OLR data from NOAA (https://psl.noaa.gov/data/gridded/data.olrcdr.interp.html) are available online. The OMI index is also available online (https://www.psl.noaa.gov/mjo/mjoindex/omi.1x.txt). The data that support the results of this study are available at https://zenodo.org/record/6979203 and from the corresponding author upon request.

## Code availability

The code for the analysis in this paper is available at https://zenodo.org/record/6979203.
