# Author Correction: Tropical modulation of East Asia air pollution

**DOI:** 10.1038/s41467-022-34315-4

**Published:** 2022-10-31

**Authors:** Myung-Il Jung, Seok-Woo Son, Hyemi Kim, Deliang Chen

**Affiliations:** 1 School of Earth and Environmental Sciences, Seoul National University, Seoul, South Korea; 2 School of Marine and Atmospheric Sciences, Stony Brook University, Stony Brook, New York, NY USA; 3 Department of Earth Sciences, University of Gothenburg, Gothenburg, Sweden

**Keywords:** Atmospheric dynamics, Environmental impact

**Correction to**: *Nature Communications* 10.1038/s41467-022-33281-1, published online 23 September 2022

The original version of this Article contained an error in Fig. 5e, where the pattern is incorrect and the black dots indicating statistical significance are missing. This has been corrected in both the PDF and HTML versions of the Article.

