# Supplementary material for: Tropical modulation of East Asia air pollution
Source: Nat Commun. 2022 Sep 23;13:5580. doi: 10.1038/s41467-022-33281-1 (PMC9508329; doi:10.1038/s41467-022-33281-1)
Supplement: Supplementary file 1 — Supplementary Information [file 41467_2022_33281_MOESM1_ESM.docx]

**Supplementary information for**

**Tropical modulation of East Asia air pollution**

**Myung-Il Jung^a^, Seok-Woo Son^a,^*, Hyemi Kim^b^, and Deliang Chen^c^**

^a^School of Earth and Environmental Sciences, Seoul National University, Seoul, South Korea

^b^School of Marine and Atmospheric Sciences, Stony Brook University, Stony Brook, New York, USA

^c^Department of Earth Sciences, University of Gothenburg, Gothenburg, Sweden

Including:

Supplementary Fig. 1

Supplementary Fig. 2

Supplementary Fig. 3

Supplementary Fig. 4

Supplementary Fig. 5

Supplementary Fig. 6

*Corresponding author: Seok-Woo Son, School of Earth and Environmental Sciences, Seoul National University, 1 Gwanak-ro, Gwanak-gu, Seoul, 08826, South Korea

E-mail: seokwooson@snu.ac.kr


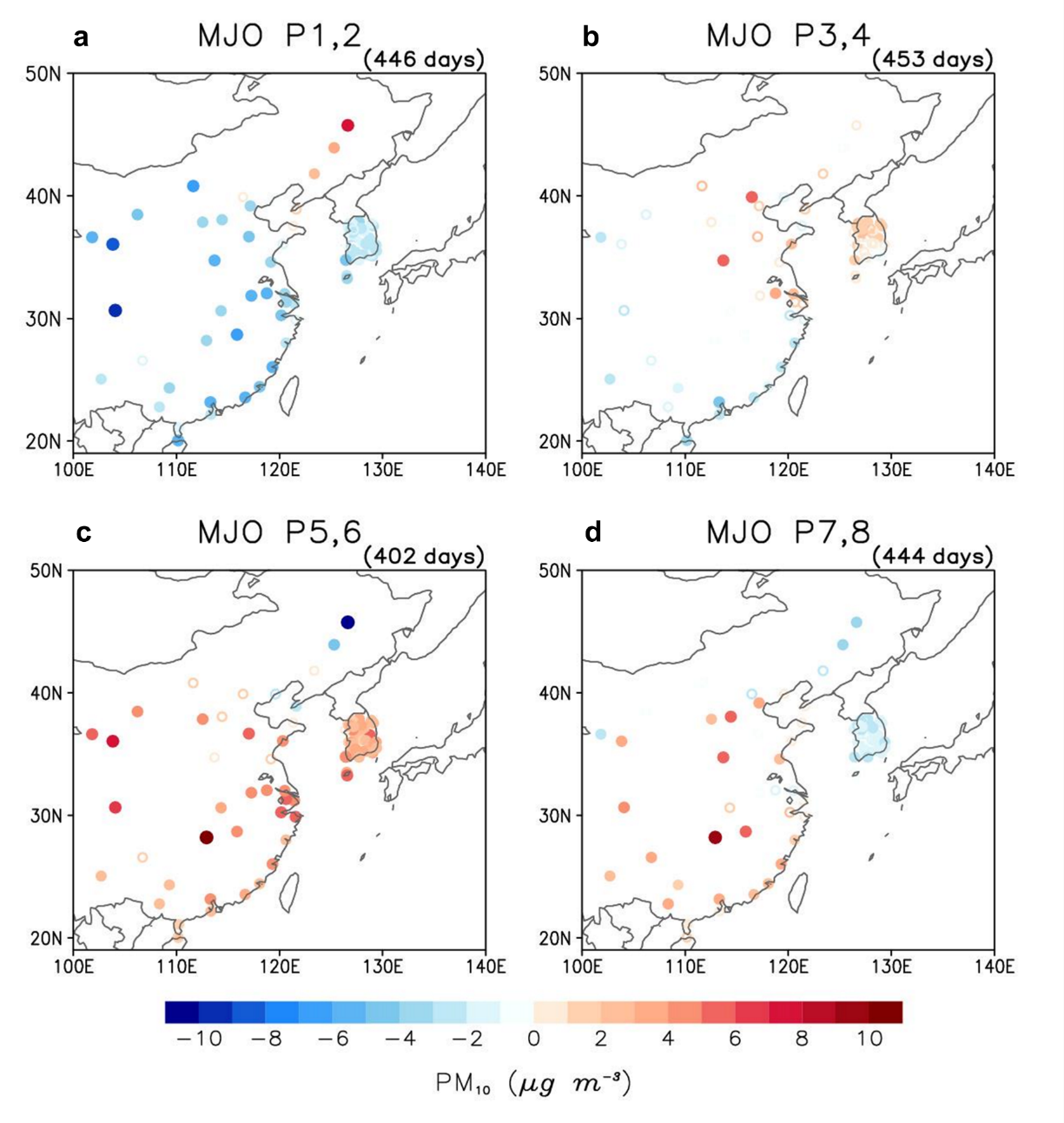


**Supplementary Fig. 1:** **Madden–Julian Oscillation (MJO)-related PM­_10_ anomalies.** Composite PM_10_ anomalies averaged over lag 6–10 days during Madden–Julian Oscillation (MJO) phases (**a**) 1–2, (**b**) 3–4, (**c**) 5–6, and (**d**) 7–8.


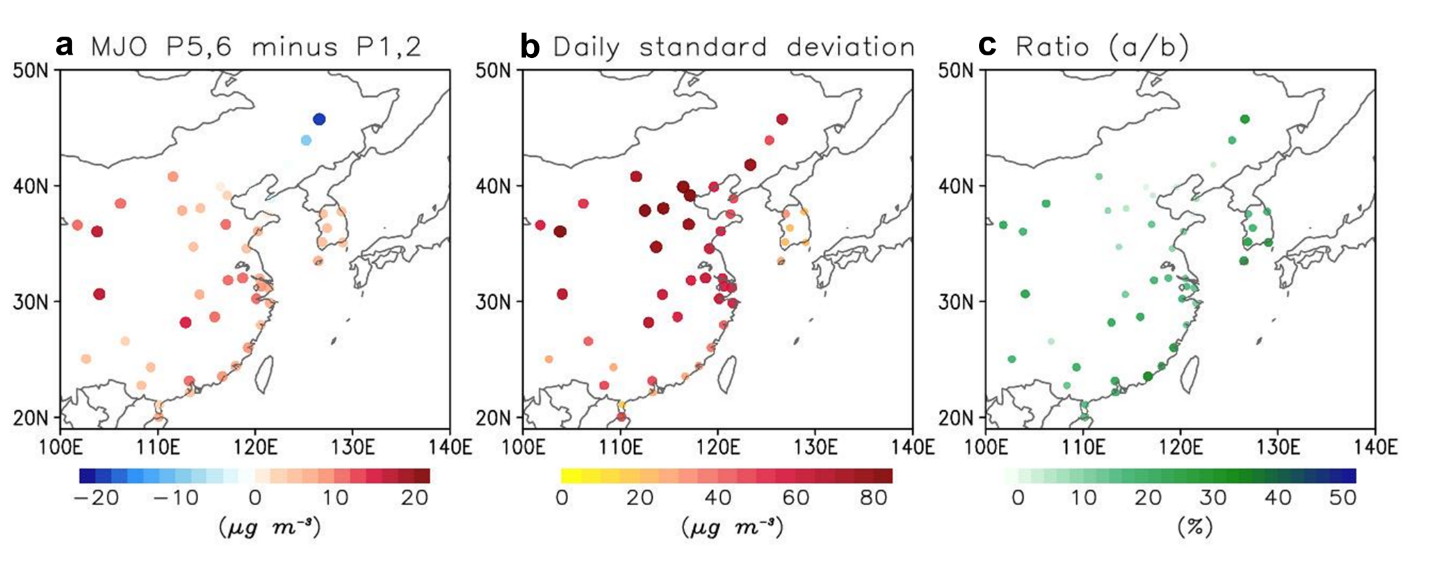


**Supplementary Fig. 2: Daily PM_10_ variability.** (**a**) Difference of PM_10_ anomalies between Madden–Julian Oscillation (MJO) phases 1–2 and 5–6, (**b**) daily standard deviation, and (**c**) their ratio.

**
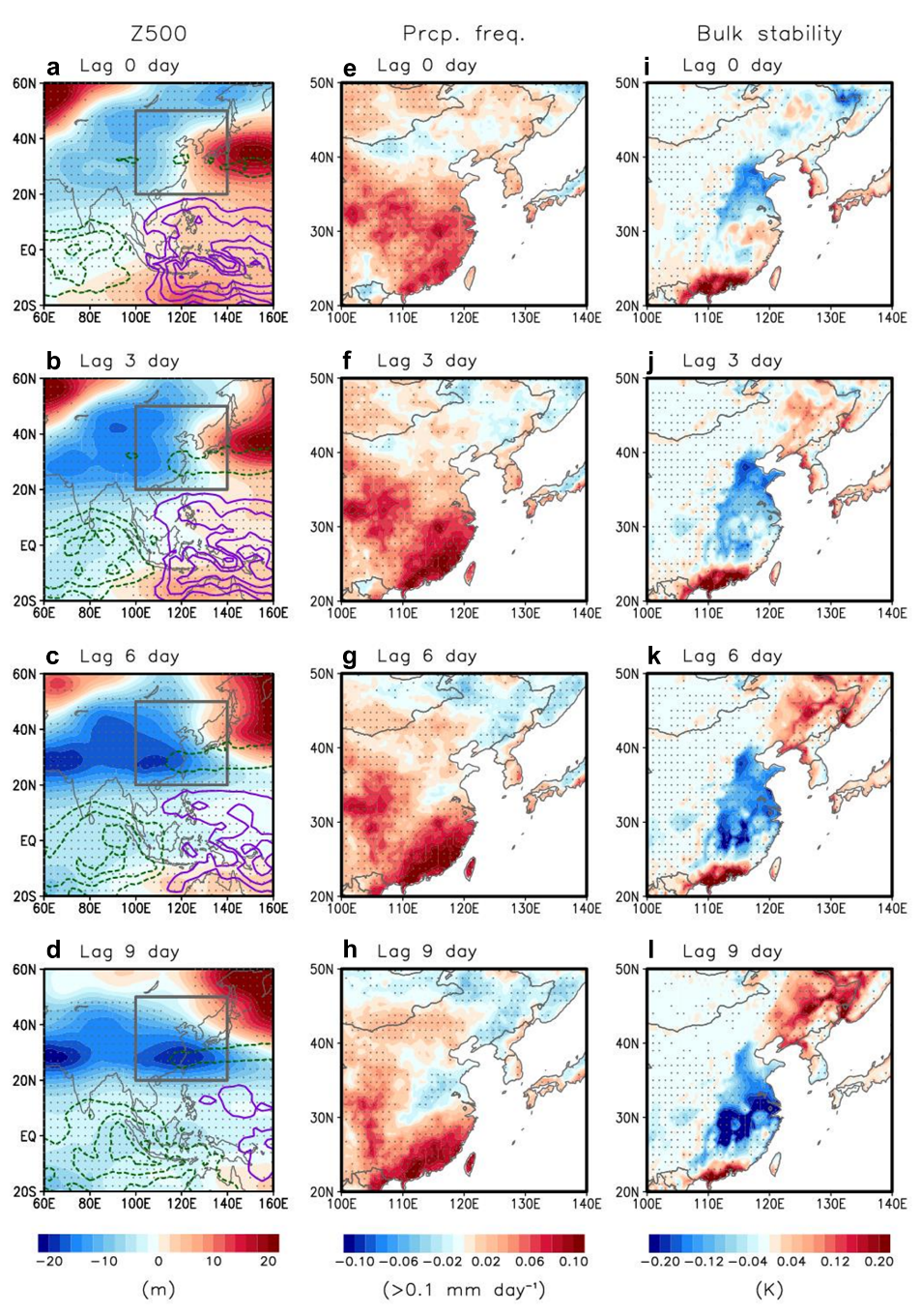
**

**Supplementary Fig. 3: Temporal evolution of atmospheric anomalies during Madden–Julian Oscillation (MJO) phase 1–2.** Composite anomalies of (**a–d**) 500-hPa geopotential height (Z500) (shading) and outgoing longwave radiation (OLR) (purple/green contours for positive/negative values with 5 W m^-2^ interval), (**e–h**) precipitation frequency, and (**i–l**) bulk stability at lag 0, 3, 6, and 9 days during Madden–Julian Oscillation (MJO) phase 1–2. Statistically significant values at the 95% confidence level are dotted. The gray box in the left column is the analysis domain used in the middle and right columns.


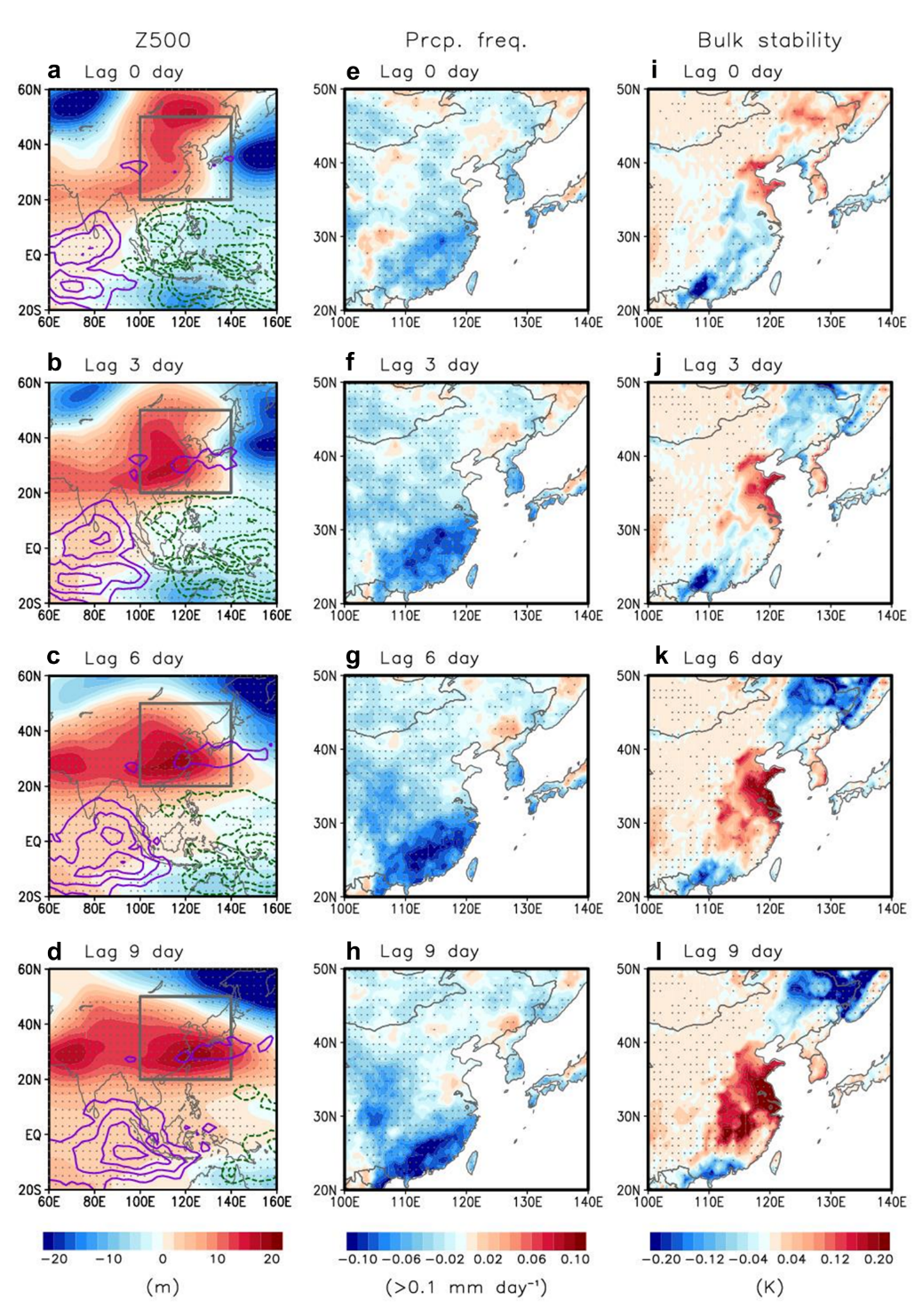


**Supplementary Fig. 4: Temporal evolution of atmospheric anomalies during Madden–Julian Oscillation (MJO) phase 5–6.** Composite anomalies of (**a–d**) 500-hPa geopotential height (Z500) (shading) and outgoing longwave radiation (OLR) (purple/green contours for positive/negative values with 5 W m^-2^ interval), (**e–h**) precipitation frequency, and (**i–l**) bulk stability at lag 0, 3, 6, and 9 days during Madden–Julian Oscillation (MJO) phase 5–6. Statistically significant values at the 95% confidence level are dotted. The gray box in the left column is the analysis domain used in the middle and right columns.


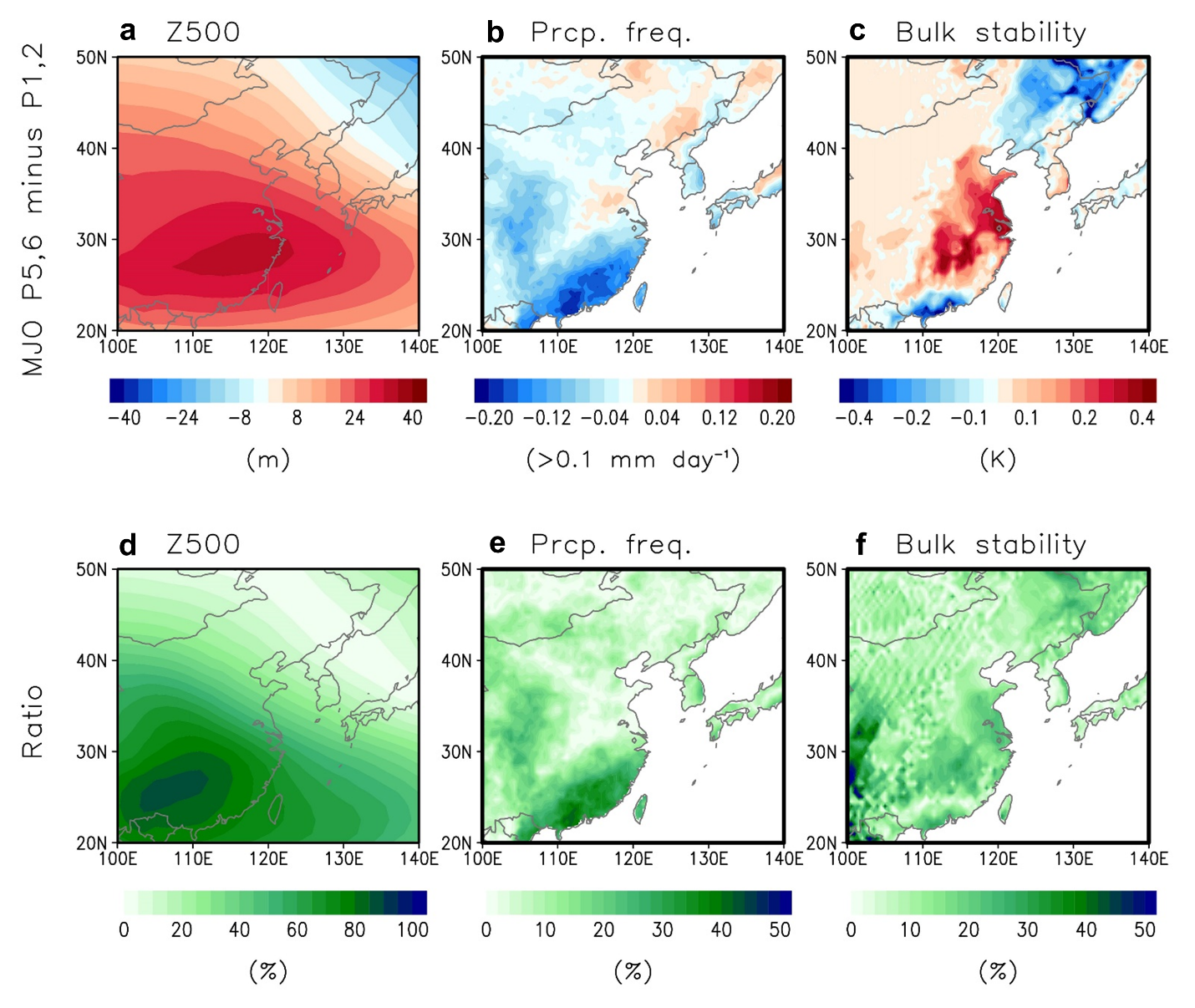


**Supplementary Fig. 5: Madden–Julian Oscillation (MJO)-related atmospheric anomalies in winter.** Difference of (**a**) 500-hPa geopotential height (Z500), (**b**) precipitation frequency, and (**c**) bulk stability anomalies between Madden–Julian Oscillation (MJO) phases 1–2 and 5–6 shown in Fig. 4. (**d–f**) Their ratio to the daily standard deviation.

**
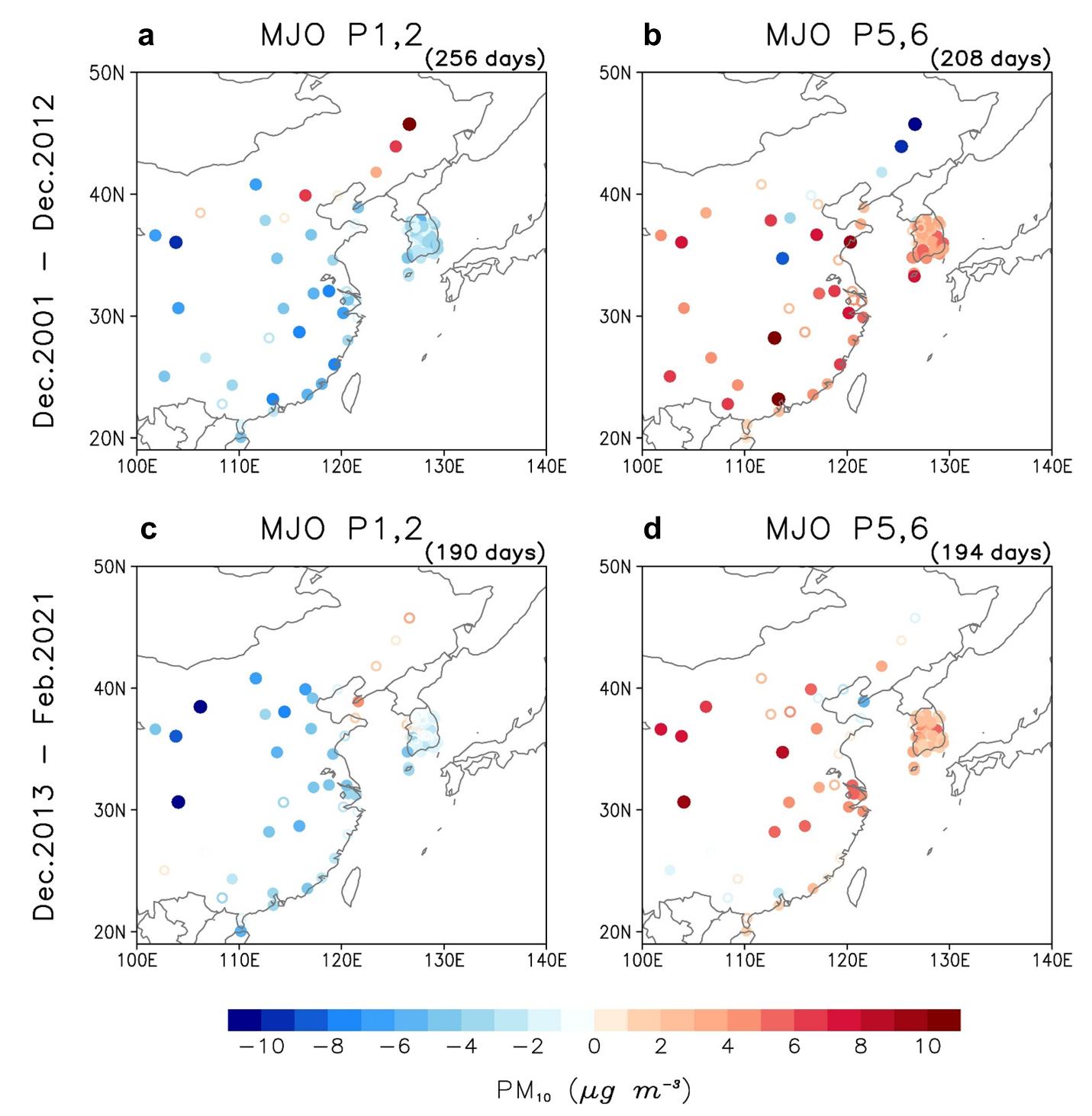
**

**Supplementary Fig. 6: Madden–Julian Oscillation (MJO)-related PM_10_ anomalies.** Composite PM_10_ anomalies averaged over lag 6–10 days during Madden–Julian Oscillation (MJO) phases 1–2 and 5–6 using (**a**, **b**) API-derived PM_10_ concentration from December 2001 to December 2012 and (**c**, **d**) AQI-collected PM_10_ concentration from December 2013 to February 2021.
